# Contribution of IgG avidity and PCR for the early diagnosis of toxoplasmosis in pregnant women from the North-Eastern region of Algeria

**DOI:** 10.4314/ahs.v17i3.7

**Published:** 2017-09

**Authors:** Hajira Berredjem, Hayette Aouras, Meriem Benlaifa, Imène Becheker, Mohamed Reda Djebar

**Affiliations:** 1 Department of Biochemistry, Faculty of Sciences, University of Badji Mokhtar, Annaba, Algeria; 2 Laboratory of Cellular Toxicology, Faculty of Sciences, University of Badji Mokhtar, Annaba, Algeria; 3 Service of Gynecology, EHS Abdallah Nouaouria Hospital, El Bouni-Annaba, Algeria

**Keywords:** Toxoplasmosis, pregnant women, serology, IgG avidity, PCR

## Abstract

**Background:**

Acute toxoplasmosis in pregnant women presents a high risk of *Toxoplasma* transmission to the fetus. Early diagnosis is difficult, especially when serological testing for IgG/IgM antibodies fail to differentiate between a recent and a past infection. In this case, we rely on IgG avidity or PCR assays.

**Objectives:**

The aim of this study was to compare conventional ELISA and IgG avidity, with PCR using B1 and P30 primers for the early diagnosis of toxoplasmosis in pregnant women.

**Methods:**

Sera were collected from 143 pregnant women and measured by ELISA for anti-*Toxoplasma* IgG, IgM, IgA and IgG avidity. DNA was extracted from 57 peripheral blood and 14 amniotic fluid samples for PCR amplification.

**Results:**

A total of 57 out 143 women were seropositive: 30 (52.6%) were IgG+/IgM− and 27 (43.8%) were IgG+/IgM+; IgA antibodies were positive in 7 (12.2%) cases. IgG avidity was low in 9 women suggesting an acute infection; 3 women presented an intermediate avidity. PCR detected *Toxoplasma* DNA in 9 women presenting low avidity and was negative for the intermediate avidity cases.

**Conclusion:**

PCR combined to avidity IgG performed better than ELISA IgG, IgM and/or IgA assays alone. PCR was useful in the case of intermediate avidity.

## Introduction

Toxoplasmosis, caused by the intracellular parasite *Toxoplasma gondii* (*T gondii*), is one of the most prevalent diseases in humans^1^. This disease is generally asymptomatic in immunocompetent human host^2^. However, *T gondii* causes substantial morbidity and mortality in immunocompromised patients^3^,^4^ and in congenitally infected infants^5^. Infection acquired by pregnant women immediately before or during gestation and its transmission to the fetus continues to be the cause of tragic yet preventable diseases, which may be more or less severe depending on the date of transmission^6^. Congenital infection acquired during the early stages of pregnancy often results in severe fetal signs such as severe neurological damages, or fetal death. Infection acquired later, within the second or third trimester, is more likely to be asymptomatic at birth leading usually to much less severe injury of the newborn and later of the child^7^. A rapid and accurate diagnosis is required in order to start the relatively efficient anti-parasitic treatment^8^.

Current diagnosis of toxoplasmosis relies mainly on serological detection of specific IgG and IgM, on fibroblast cell culture or mice inoculation of amniotic fluid or fetal blood^3^,^9^. The presence of IgM is, in general, an indication that the host has recently been infected. However, these antibodies can persist for months or even years after acute infection. Consequently, the concomitant presence of IgM and IgG does not always indicate an acute infection, so it is necessary to study antibody kinetics based on a serological control 2–3 weeks later^10^. Moreover, serological testing may fail during the active phase of *T. gondii* infection because the antibodies titres are low; therefore, the high risk of congenital toxoplasmosis of a fetus may be undetected because the pregnant mother might test negative during the active phase of *T. gondii* infection. When high levels of IgG antibodies are present in sera, serological testing does not distinguish a recent infection from one acquired a longtime before and detection of specific IgM response cannot help determine if infection was recent^11^. This inconvenience has limited the use of the serological tests. More studies have shown that IgG avidity assay^10^,^14^,^15^ or polymerase chain reaction (PCR)^12^,^13^ are useful for identification or exclusion of *T. gondii* acute infection, or for estimation at the time of seroconversion.

In this study, IgG avidity and PCR have been evaluated, on the basis of the results obtained with conventional serological tests, as a diagnostic tool for the diagnosis of toxoplasmosis in Algerian pregnant women, and for the descrimination between acute and chronic infection.

## Patients and methods

This prospective study was carried out at Abdallah Nouaouria Hospital, El Bouni-Annaba. Peripheral blood (PBL) and amniotic fluid (AF) samples were collected during diagnosis for toxoplasmosis in 143 pregnant women, between 22 and 43 years old. None of the mothers had apparent symptoms of toxoplasmosis during their pregnancy and no antibiotic drugs were previously prescribed to the patients. Two blood samples, with respectively an interval of 21 days, were taken from each patient. Therefore, a total of 286 sera samples were checked for anti-*Toxoplasma* antibodies by serological tests : IgG, IgM and IgA ELISA assay. A total of 57 samples (one sample from each patient) with a positive anti-*Toxoplasma* IgG were further analysed using the IgG avidity. Blood and AF samples were taken for DNA extraction. Gene amplification was processed on 57 PBL samples; PCR on AF was done to 14 women suspected to have acute infection which can lead to a congenital toxoplasmosis. This study was approved by the Hospital Ethical Committee, and informed consent was obtained from all the patients.

## IgG, IgM and IgA ELISA

All patients' sera were tested for the presence of specific IgG, IgM and IgA antibodies to *Toxoplasma* using an ELISA kits (Platélia Toxo, Diagnostic Pasteur, France) according to the manufacturer's instructions. The anti-*Toxoplasma* IgG, expressed in IU, were calculated from a standard curve. « Double sandwich » ELISA was used for the detection of IgM and IgA antibodies. Sera samples were processed in duplicate.

## IgG avidity

The *Toxoplasma*-specific IgG avidity assay was performed by ELISA (SFRI® laboratoire, France) to discriminate between acute and chronic infection. A total of 57 sera samples were diluted 1/200 and added (100 µl/well) on 2 rows of a plate (row A and row B). After 45 min of incubation at 37°C, the row B was washed 3 times with the modified PBST buffer containing 6 M urea and 4 times with PBST containing 0.05% Tween 20 in order to remove low-avidity antibodies from their binding sites. The control row A was washed 3 times with buffer without urea. The anti-human IgG conjugated with horseradish peroxidase (Dako Glostrup, Denmark) was added with the dilution of 1/1000 in PBST. After incubation and washing, the chromogenic substrate, O-phenylenediamine (Merk, Darmstadt, Germany), was added. The reaction was stopped by addition of sulfuric acid 20%. The absorbance was read at 492 nm. The IgG avidity index (AI: %) was calculated on the basis of the formula : AI = Abs (PBS-urea)/Abs(PBST) ×100.

Three different avidity classes were determined: AIs lower than 25%, between 25% and 35%, and above 35% were classified as low, intermediate, and high avidity, respectively.

## DNA parasite preparation

DNA extracted from the tachyzoites of *T. gondii*-RH strain (Institut Pasteur, Tunis) was used as positive control for PCR. The parasites were harvested from the peritoneal exudate of Swiss-Webster female mouse intraperitoneally infected 3 days earlier. The DNA parasite was extracted using the salting out method. The concentration of purified DNA was mesured by UV spectrophotometry (GENEQUANT, pharmacia Biotech). The DNA samples were stored at −20°C.

## Polymerase chain reaction of *T. gondii* B1 and P30 genes

PBL and AF DNA were extracted by the salting out method using the Sodium Chlorure 6 M and precipitated with ethanol^16^. The DNA pellet was dissolved in TE buffer and concentrations were then determined.

PCR amplification was carried out in two separate assays : nested PCR, using the 35-fold repetitive DNA region B1 of *T. gondii* primers sets and conventional PCR-ELISA using the major surface antigen P30 gene primers sets. The primer sequences and the expected size of B1 and P30 genes PCR products are shown in Table 1.

**Table 1:** B1 and P30 Genes primer sequences for the amplification of *T.gondii* DNA.

Target Gene	Primers	Sequence (5′ → 3′)	Positions	Size (bp)
**B1** **(Nested** **PCR)**	Outer sense strand Outer nonsense strand Inner sense strand Inner nonsense strand	**Tx2 :** TCT TTA AAG CGT TCG TGG TC **Tx4 :** GGA ACT GCA TCC GTT CAT GAG **Tx1 :** GGC GAC CAA TCT GCG AAT ACA ACC **Tx3 :** TGC ATA GGT TGC AGT CAC TG	887 - 868 694 - 714 853 - 831 757 - 776	193 96
**P30** **(PCR-ELISA)**	Sense strand Nonsense strand	**GBI5 :** AGC TGG TGG ACG GGG GAT TC **GBI6 :** GTC TGC ACC GTA GGA GCA CC)	689 - 709 875 - 895	126

For nested PCR using the B1 gene, previously described by Burg^17^, amplification was carried out in a 50 µl reaction mixture containing 10 mM Tris-HCl (pH 9.0), 50 mM KCl, 1.5 mM MgCl_2_ (Promega, France), 0.2 mM of each dNTP (Bohringer Mennheim, Germany), 50 pmol of each primer (Diasorin, Biomedica, Italy), 2.5 U of Taq DNA polymerase (Promega, France) and DNA extracted from each sample. Reactions were run in a Perkin-Elmer thermocycler by using a step cycle program. After initial denaturation of the DNA at 94°C for 5 min, 40 cycles were run : 60 s with a denaturing temperature of 94°C, 60 s with an annealing temperature of 55°C, and 90 s with an extension temperature of 72°C, with a 5 min 72°C extension after the 40 cycles. After the first 40 cycles, 10 µl of each sample was transferred to a second PCR reaction into 40 µl of fresh mixture containing the inner primers pair and the procedure was repeated for 35 cycles.

The conventional PCR-ELISA was assessed for the amplification of the P30 gene18 using specific primers and under the same conditions as B1 gene for 40 cycles.

Each amplification run included negative and positive controls. All samples were tested in duplicate. Amplicons were revealed by ethidium bromide on 2% agarose gel electrophoresis, and then visualized under UV illumination.

For P30 gene, detection of hybrids was performed by an ELISA-PCR kit (GEN-ETI-K, DEIA, Diasorin, Italy) as described by the manufacturer. The solid phase was prepared in advance : the wells of a microplate were coated with biotinylated DNA probe (5′ TCCCTT GATGCAACCGACCACAAA). A result was considered positive when one reaction yielded an amplification product hybridizing to the specific DNA probe.

## Results

### Antibody detection

According to respective levels of anti-*Toxoplasma* IgG and IgM in the first positive sample and in the second one, a *Toxoplasma* seropositive status during pregnancy was defined in 57 (39.8%) women. Table 2 shows the huge diversity of the techniques used and presents data on the levels of antibody detection.

**Table 2:** Serological (ELISA) and molecular (PCR) results from 57 *Toxoplasma* seropositifs pregnant women

Patient N°	Gestational Age (weeks)	Anti-*Toxoplasma* antibodies					DNA amplification (duplicate assays results)			
							PCR-ELISA (P30 gene)		Nested PCR (B1 gene)	
		IgA	IgM	IgG (IU)	IgG Avidity (%)	Diagnosis	Blood	AF	Blood	AF
1	16	+	+	220	18	AI	+/−	−/−	+/+	−/−
2	16	−	+	>240	63	CI	−/−		−/−	
3	20	−	−	>240	67	CI	−/−		−/−	
4	16	+	+	>240	15	AI	+/+	−/−	+/+	−/−
5	15	−	+	>240	71	CI	−/−	−/−	−/−	−/−
6	16	−	+	>240	65	CI	−/−		−/−	
7	8	−	+	>240	20	AI	+/+		+/+	
8	19	−	+	>240	58	CI	−/−		−/−	
9	12	−	−	>240	47	CI	−/−		−/−	
10	12	−	−	>240	62	CI	−/−		−/−	
11	16	−	+	>240	55	CI	−/−		−/−	
12	13	−	−	>240	49	CI	−/−		−/−	
13	16	−	+	114	44	CI	−/−	−/−	−/−	−/−
14	14	−	+	>240	28	IA	−/−	−/−	−/−	−/−
15	15	+	+	>240	21	AI	+/+	+/+	+/+	+/+
16	8	−	+	129	70	CI	−/−			
17	16	−	−	80	59	CI	−/−		−/−	
18	6	−	−	>240	65	CI	−/−		−/−	
19	16	−	−	110	51	CI	−/−		−/−	
20	16	−	−	>240	53	CI	−/−		−/−	
21	12	−	+	>240	48	CI	−/−		−/−	
22	8	−	+	125	47	CI	−/−		−/−	
23	13	−	−	110	52	CI	−/−		−/−	
24	15	−	+	180	31	IA	−/−	−/−	−/−	−/−
25	16	−	+	240	68	CI	−/−	−/−	−/−	−/−
26	14	−	−	80	60	CI	−/−		−/−	
27	10	+	+	240	14	AI	+/+		+/+	
28	8	−	−	112	52	CI	−/−		−/−	
29	15	+	+	>240	16	AI	+/+	+/+	+/+	+/+
30	16	−	−	>240	42	CI	−/−		−/−	
31	12	−	−	180	47	CI	−/−		−/−	
32	14	−	−	220	51	CI	−/−		−/−	
33	14	−	+	>240	68	CI	−/−		−/−	
34	16	+	+	>240	20	AI	+/+	+/−	+/+	+/+
35	13	−	−	>240	43	CI	−/−		−/−	
36	15	−	+	>240	44	CI	−/−	−/−	−/−	−/−
37	11	−	−	220	71	CI	−/−		−/−	
38	13	−	−	240	65	CI	−/−		−/−	
39	18	−	−	>240	49	CI	−/−		−/−	
40	12	−	−	118	46	CI	−/−		−/−	
41	16	−	−	200	29	IA	−/−	−/−	−/−	−/−
42	12	−	+	180	39	CI	−/−		−/−	
43	15	−	+	>240	19	AI	+/+	−/−	+/+	−/−
44	10	−	−	>240	61	CI	−/−			
45	14	−	−	>240	42	CI	−/−		−/−	
46	11	−	−	140	43	CI	−/−		−/−	
47	8	−	−	220	52	CI	−/−		−/−	
48	10	−	+	240	41	CI	−/−		−/−	
49	9	−	−	>240	42	CI	−/−		−/−	
50	11	−	−	>240	52	CI	−/−		−/−	
51	12	−	+	220	46	CI	−/−		−/−	
52	12	−	−	>240	44	CI	−/−		−/−	
53	15	−	−	240	46	CI	−/−		−/−	
54	16	+	+	114	20	AI	+/+	+/+	+/+	+/+
55	17	−	−	>240	55	CI	−/−		−/−	
56	10	−	+	240	51	CI	−/−		−/−	
57	9	−	−	>240	50	CI	−/−		−/−	

**AI:** Acute Infection, **CI:** Chronic Infection, **IA:** Intermediate Avidity, **AF:** Amniotic Fluid, **IU:** International Unit Duplicate assays results are given for DNA amplification.

The IgG ELISA antibodies were evaluated with the following IU:between 80 and 220 in 20 women (35%) and 240 in 37 women (64.9%). For IgM antibodies, 27 (47.3%) of 57 cases tested positive with high titers. The IgA antibodies were found in 7 out of 57 (12.2%) samples (Tab. 3).

**Table 3:** Rates of *Toxoplasma* acute infection among the study pregnant women. Diagnosis was performed on blood and amniotic fluid samples.

	Serological Results						PCR Results								
							B1 gene				P30 gene				

Trimester	IgA		IgM		Low Avidity		Blood		AF		Blood		AF		Diagnosis (AI)	
	Nbr	(%)	Nbr	(%)	Nbr	(%)	Nbr	(%)	Nbr	(%)	Nbr	(%)	Nbr	(%)	Nbr	(%)
First	1/57	(1.75)	12/57	(21.05)	4/57	(7.01)	1/57	(1.75)	0/14	(0.00)	1/57	(1.75)	0/14	(0.00)	1/57	(1.75)

Second	7/57	(12.28)	15/57	(26.31)	5/57	(8.77)	8/57	(14.03)	4/14	(28.57)	8/57	(14.03)	4/14	(28.57)	8/57	(14.03)

**AF :** Amniotic Fluid

**AI :** Acute Infection

Among the 57 women, a total of 30 (52,6%) sera were IgG+/IgM−, while 27 (47.3%) sera were IgG+/IgM+. No sera sample was IgG−/IgM+.

A possible acute infection was suspected for 14 women on the basis of a combination of positive specific IgG/IgM and/or IgA, using two blood samples from each patient, with respectively an interval of 21 days.

### IgG Avidity

*Toxoplasma* IgG Avidity assay has been performed on 57 sera with IgG positive results. High avidity antibodies (indicative of chronic toxoplasmosis) were present in 45 (78.9%) women; intermediate avidity was determined for 3 (5.2%) women and low IgG avidity antibodies (indicative of acute toxoplasmosis) were found in 9 (15.7%) women, without evidence of *Toxoplasma* infection (Tab. 2). Therefore, 9 out 14 suspected infected women showed low IgG avidity which confirmed an acute infection.

Women, with positive IgM and IgA, were shown to be recently infected indicating an active toxoplasmosis infection (Tab. 2). All subjects presenting an acute toxoplasmosis were at least in the first (1 women out 9) or second (8 women out 9) trimesters of their pregnancies. Therefore, toxoplasmosis infection occurred most likely before or just after conception with a substantial risk for the fetus.

### Polymerase chain reaction assay

The PCR results are summarized in Table 2. *T.gondii* DNA was detected by PCR targeting of the B1 and P30 genes in 9 of 57 (15.7%) blood samples and 4 of 14 (28.5%) AF samples obtained from pregnant women. PCR positive results with the AF samples demonstrated a congenital toxoplasmosis. A total of 8 women out 9 were in the second trimester of their pregnancies (Tab. 3).

All the PCR positive cases were seropositive; these included 7 samples (12.2%) that were positive for IgG, IgM and IgA antibodies and 2 (3.5%) samples that were positive for both IgM and IgG antibodies. A high IgG avidity and a negative PCR results excluded recent *Toxoplasma* infection in the other women. With regard to positif PCR and low IgG avidity results, 7/9 patients have an IgG-ELISA value ≥ 240 IU and a positive IgA antibodies. Viewed separately, IgG avidity alone didn't demonstrate the acute or chronic infectious status in 3 women who showed an intermediate IgG avidity, whereas PCR gave negative signals with both gene targets and demonstrate a chronic infectious status for these women.

The PCR-ELISA targeting the P30 gene had a lowest sensitivity compared to the nested PCR targeting the B1 gene. In fact, when PBL and AF samples were used, only one assay out of two duplicate tests returned positive (patients 1 for PBL samples and patients 15 and 34 for AF). No PCR product was obtained with the negative controls in any experiment.

## Discussion

When a *T. gondii* primary infection is acquired during pregnancy, the parasite may be transmitted to the fetus. The parasite reaches the fetus transplacentally, causing various degrees of damages, depending on the virulence of the parasite, the immune response of the mother and at what trimester the infection was acquired, resulting in fetal death or in severe clinical symptoms^6^. Pregnant women are frequently asymptomatic, making a diagnosis difficult. Determination of recently acquired *T. gondii* infection in pregnant women must be made as early as possible to begin an adequate anti-parasitary treatment wich can improve the prognosis^19^.

Currently, the diagnosis of toxoplasmosis is based on immunological testing that give the titer of circulating antibodies. The presence of specific, IgM^20^ or IgA,^21^,^22^ may help to identify recent infection. However, increased levels of IgG and IgM antibodies do not distinguish a recent infection from one acquired a long time before. Therefor, a rapid and effective diagnosis is crucial to initiate an adequate treatment.

In the present study, the tests we used for clarifying the serological status of pregnant women showed IgG and/or IgM anti-*Toxoplasma* antibodies for 57 (39.8%) out 143 women, which represent the global prevalence of anti-*T gondii* IgG antibodies in this population. *Toxoplasma* infection during pregnancy was suspected among 14 women according to respective level of anti-*Toxoplasma* IgG and IgM in the first and second samples. On the other hand, results revealed higher IgA antibody titers from acutely infected pregnant women which indicates that in the presence of *Toxoplasma* specific IgG and IgM antibodies, the additional presence of IgA was sufficient to identify the acquisition of infection within the previous 8 to 16 weeks. There is an association between the high level of IgA and IgM which may indicate acute phase of toxoplasmosis. It has faster kinetics, suggesting that the infection occurred less than eight months previously^23^,^24^. IgA antibody determination seems to be important for screening of pregnant women and for prenatal diagnosis.

When using the presence of specific IgM as criteria for identifying *T. gondii* infection in early pregnancy, many women will be falsely identified as possibly infected and unnecessarily undergo diagnostic amniocentesis and anti-parasitic treatment. In fact, true positive results must be carefully interpreted^25^ as IgM antibodies might persist for years after primary infection (residual IgM)^11^,^26^. This fact has limited the use of these methods, because it is not possible to determine if the patient has an acute infection or if the infection had occurred months before^27^. Serological testing may fail during the active phase of *T. gondii* infection because the antibodies titres are low; therefore, the high risk of congenital toxoplasmosis of a fetus may be undetected because the pregnant mother might test negative during the active phase of *T. gondii* infection. The ideal situation for the diagnosis of *Toxoplasma* infection in pregnancy would be to have an antibody-negative sera sample collected before conception or at the beginning of pregnancy. However, this was not possible in this study.

In combination with the serological conventional tests, IgG avidity is an auxiliary test which permits to differentiate past and present *Toxoplasma* infection when the IgM serological reaction is positive in an asymptomatic patient^28^,^29^,^30^. In 1989, Hedman et al.^28^ introduced the IgG avidity methode for detecting recently acquired *Toxoplasma* infection, based on the strength of the binding of specific IgG to multivalent *Toxoplasma* antigen^31^ and than on the elution of low avidity antibodies by a protein-denaturing agent, mostly urea^32^,^33^. During the course of the immune response, there is maturation of antibody affinity that increases progressively over weeks or months. In crease in IgG affinity result from an antigen-driven B-cell selection process, resulting in an increase in complementarity of the antigene-antibody-binding site^34^. This binding strength was found to be low in acute phase and high in chronic phase of toxoplasmosis^35^,^36^; therefore, detection of a low IgG avidity is a reliable indicator for recent toxoplasmosis, whereas a high avidity shows that the infection occurred in the previous 4–5 months^37^,^38^. This is most useful in pregnant women in their first months of gestation who have a positive test for both IgG and IgM *Toxoplasma* antibodies^34^. In this study, recent infection in 9 pregnant women was confirmed by the low avidity index found by IgG avidity ELISA. In fact, we showed that a low avidity index associated with IgG, IgM and/or IgA seroreactivity, is a good indicator that an acute *T. gondii* infection within the last 4 months has been occured. Among 57 seropositive women, the measure of avidity on the first sera sample have allowed us to take accurate conclusions in 94.7% of cases. The antibody assay combinations and comparison between conventional ELISA and IgG avidity have proven to be very useful. Lecolier and Pucheu,^39^ concluded that the *Toxoplasma*-specific IgG avidity test should be performed with the first blood sample collected in early pregnancy to exclude acute infection during gestation.

For three patients the avidity index showed a border line result. When avidity is low or borderline it may be misleading and a more careful interpretation is critical. Low-avidity results may persist for as long as 1 year and should not be considered as a sufficient argument to confirm a recent seroconversion and even less to prescribe invasive procedures like amniocentesis^34^. In these cases direct detection of the parasite is necessary for a definitive diagnosis.

The use of the PCR test enable the rapid detection of specific *Toxoplasma* genes and its combination with serological tests carried out on PBL and AF, facilitate earlier diagnosis of congenital toxoplasmosis. Among the many factors influencing the PCR outcome, the choice of the DNA target and primers is generally considered as essential. Few DNA target loci have been described for *Toxoplasma* PCR, but different primer pairs have been used in different assays. The principal gene target remains the 35-fold repetitive and conserved B1 gene. The detection level of PCR is 1 to 10 parasites in the presence of 10^5^ human leucocytes^17^. In our study, *T. gondii* DNA could be detected in 9 women who showed positive IgG and IgM antibodies as well as low IgG avidity suggesting that these women may have acquired infection during pregnanacy. With regards to the PCR assay by the use of PBL and AF, both B1 and P30 primer sets performed quite equally well and therefore appear adequate for *Toxoplasma* identification; but, with primer set P30, an additional step (achieved through an ELISA-PCR assay) for confirming the identity of the PCR product is of utter importance. However, B1 gene proved valuable PCR for *T. gondii* detection better than P30 gene. Our results showing 3 false negative PCR (Tab. 2), are in agreement with those of Bastien^40^ who validate the current choice targeting B1 gene, which appear clearly more sensitive than assays targeting the single copy P30 gene.

The PCR results were in concordance with those of IgG avidity test. Moreover, PCR was very helpful in cases of intermediat avidity. 3 cases were IgG avidity borderline but PCR negative.

Based on the results of this study, the IgG avidity and the PCR tests performed better than the conventional ELISA for the diagnosis of *Toxoplasma* infection in pregnant women.

When only conventional ELISA was used, 24.5% (14 of 57) acute infections were suspected. When ELISA and IgG avidity were combined, 3 suspected infections out of 14 (05.2%) yielded an intermediat avidity. When ELISA, IgG avidity, and PCR findings were combined, the acute toxoplasmosis of pregnant women could be demon strated in 15.7% of cases (9 of 57). This study confirms the complementary need of the techniques used for the screening of pregnant women, but some authors recommend PCR than most serologic techniques^13^

## Conclusion

In this study, the conventional serodiagnosis of toxoplasmosis, on the basis of the first and the second sera sample, was sufficient to determine the serological status of most women and the demonstration of IgA antibody appears useful for diagnosis of *Toxoplasma* infection. However, this is was not possible for some women. The IgG avidity assay has been demonstrated to have the utility to exclude recent infection in early pregnancy for women who otherwise, on the basis of a positive specific IgM result, would have been identified as having a recent infection. From these results it can be concluded that IgG avidity, performed in single samples from positive anti-*Toxoplasma* IgM pregnant women, is a valuable diagnostic tool. Overmore, single PCR-negative samples in conjunction with an IgG/IgM positive test result could confirm a past infection in the presence of serologic results that are difficult to interprete, especially for intermediate avidity.

The main finding of our study was the significant association between high IgG anti-*T. gondii* titers, low index avidity and PCR detection of *T. gondii* DNA and the diagnosis of acute toxoplasmosis. Moreover, the molecular biology technique was useful in pregnant women with seroconversion, to exclude the presence of *T. gondii* DNA in AF. These women will be saved from unnecessary anxiety, additional examinations and treatment. However, to the best of our knowledge, no studies have been published using both IgG avidity and PCR for the diagnosis of toxoplasmosis on pregnant women in Algeria. We hope that our study will open new perspectives for the systematic introduction of these techniques in the diagnosis of toxoplasmosis.
